# Peer Review of “Mothers’ Knowledge of and Practices Toward Oral Hygiene of Children Aged 5-9 Years in Bangladesh: Cross-Sectional Study”

**DOI:** 10.2196/70144

**Published:** 2025-02-03

**Authors:** Md Hafizul Islam

**Affiliations:** 1 Institute of Nutrition and Food Science, University of Dhaka, Dhaka, Bangladesh, 880 000000000

**Keywords:** mothers’ knowledge and practices, oral hygiene, child oral health, Bangladesh

*This is the peer-review report for “Mothers’ Knowledge of and Practices Toward Oral Hygiene of Children Aged 5-9 Years in Bangladesh: Cross-Sectional Study.”*

## Round 1 Review

This is an interesting piece of research [1], which highlights mothers’ knowledge and practices regarding their children’s oral health in Dhaka City. However, several issues made the study scientifically questionable. The major issues are as follows. The study included mothers from two hospitals in Dhaka City, but the title of the study does not mention this. The sample selection from the mothers visiting the hospitals might not represent general mothers from the whole of Dhaka. Thus, this study might not be generalizable to all mothers in Dhaka City.

### Introduction

Revise the last paragraph of the Introduction to highlight the study gap in Bangladesh and clearly state the objective of the study. Use the formal word “mother” and avoid the word “moms.”

### Methods

#### Study Setting and Participants

Give clear reasoning as to why you selected study participants from the hospitals. The last line is confusing. It is not clear whether the participants filled out the questionnaire on their own or they were interviewed by the enumerators.

#### Sampling Technique

Please mention the nonresponse bias for the convenient sampling. Give a short description of the pretesting mentioning the number of samples, period, and location for it.

#### Measurement of Knowledge and Practice Score

Give the 15 knowledge-related questions and 13 practice-related questions in the supplementary file. Mention if these questions are your own or if you used any valid tools or questions adopted from the relevant previous studies. Give adequate information regarding the scoring system of the variables, mentioning the highest possible aggregated score and examples of two questions (one for knowledge and one for practice).

#### Statistical Analyses

The authors mentioned that they used the Mann-Whitney *U* test and the Kruskal-Wallis test. However, they did not mention the underlying assumptions of the tests. Moreover, the Results section also shows the *χ*^2^ test but is not mentioned in the Methods section. Furthermore, the last line of the Results of the abstract shows the Pearson correlation coefficient, but nothing is mentioned in the Methods or Results section of the entire manuscript.

### Results

#### Table 1

It is confusing as the text description of Table 1 and the title of Table 1 are different. It is recommended to use two separate tables: one for socioeconomic variables and another for the frequency distribution of the knowledge level among socioeconomic variables. Mention the knowledge- and practice-related raw scores first and then the cross-tab results. There is a major mistake in the results of Tables 1 and 2. The frequency distribution for educational status, occupation, family type, number of family members, and monthly income in Tables 1 and 2 are the same. However, the *P* values are different. How is this possible? Please check the results.

### Discussion

It is confusing whether the practice was for the children or how a mother takes care of their children’s dental health. Mention the implications of your findings rather than just comparing the findings with previous studies. State the limitation of the study, especially the bias regarding convenient sampling. Provide a section on the public health significance of the study findings in Bangladesh.

### Conclusion

The Conclusion section of the study is poorly written and not focused on the findings of the study. Revise the Conclusion section to highlight your study findings.

## Round 2 Review

The authors impressively amended the initial version of the manuscript based on the reviewers’ comments. However, several issues remain unaddressed.

1. The authors should include the city in the title of the study. You can revise the title to “Knowledge and practices towards oral hygiene of children aged 5‐9 years old: a cross-sectional study among mothers visited tertiary level hospitals in Dhaka, Bangladesh.”
2. Use the full form when it appears first and then use the abbreviation afterward. For example, “KP” in the abstract.
3. Please mention this statistical test in the Methods section of the abstract. You did not mention the *χ*^2^ test and Pearson correlation.
4. It is recommended to make the recommendation simple and easy to understand for the readers. Avoid duplication of the same term.
5. In the sample size calculation, you used *P*=.58 and *P*=.57. Please clarify why you used those prevalences. Cite the relevant study here.
6. Before the heading for the sociodemographic variables in the Methods section, you mention outcome measures. However, the sociodemographic variables are not your outcome variables according to your objectives. You can remove the term outcome measures from here.
7. You mentioned that you used 13 questions for the assessment of practices. Thus, according to your scoring approach, there should be a score of 1-13, but here, it is 1-11.
8. Please mention the name of the software and version you used for the statistical analysis.
9. Revise the sentence before Table 1. You can make it two sentences. One for family income and another for occupation.
10. There is no chi-square–related data in Table 1. Please remove the footnotes from Table 1.
11. In Figure 1, it is recommended to keep the values to one decimal point for 1a and 1b.
12. Please revise the sentence before Table 3 to give a clear meaning.
13. You can remove the percentage symbol from the value and give it in the vertical axis title.
14. Please give the correlation results in the main manuscript or as a supplementary table.
15. The authors overlooked the association of knowledge and practice with income and family size. Please give more details on those two points in the Discussion section.
